# Draft Genome Sequence of Lactococcus lactis Strain 12-16-PSH, Isolated from Prostokvasha from the Arkhangelsk Region of Russia

**DOI:** 10.1128/mra.01265-22

**Published:** 2023-02-22

**Authors:** Alexander G. Elcheninov, Kseniya S. Zayulina, Polina A. Shcherbakova, Mariia K. Kremneva, Liliya A. Gavirova, Andrey I. Shestakov, Ilya V. Kublanov

**Affiliations:** a Winogradsky Institute of Microbiology, Federal Research Center of Biotechnology, Russian Academy of Sciences, Moscow, Russia; b Microbiology Department, Faculty of Biology, Lomonosov Moscow State University, Moscow, Russia; University of Maryland School of Medicine

## Abstract

A draft genome sequence of Lactococcus lactis strain 12-16-PSH, which was isolated from prostokvasha, is reported. The genome assembly of strain 12-16-PSH contained 63 contigs, with a total length of 2,468,647 bp. A total of 2,421 protein-coding genes were predicted, among which 6 encoded bacteriocins while 15 encoded glycosyl transferases, presumably involved in exopolysaccharide biosynthesis.

## ANNOUNCEMENT

There is a great variety of fermented milk products (FMPs), which depend on microbiome composition, production technology, and milk source. Lactic acid bacteria play a crucial role in milk fermentation, being one of the major components of FMPs (1). The genome sequence of strain 12-16-PSH, which was isolated from prostokvasha (an FMP) from the Arkhangelsk region of Russia (62°01′41″N, 45°10′23″E) that had been prepared from Kholmogor-breed cow milk (sample 16 PS [2]), is reported. Strain 12-16-PSH was isolated using serial dilutions, followed by cultivation on MRS agar medium (HiMedia) under anaerobic conditions using a BBL GasPak 150 anaerobic system with a BBL GasPak Plus envelope (BD) at 32°C.

For genomic sequencing, strain 12-16-PSH was cultivated on MRS agar medium under aerobic conditions for 72 h. The grown culture was mixed with 500 μl TSE buffer (100 mM Tris-HCl, 100 mM EDTA, 150 mM NaCl [pH 8.0]). Genomic DNA was isolated using the FastDNA spin kit for soil (MP Bio), and DNA libraries were prepared using the KAPA HyperPlus kit (Kapa Biosystems) according to the manufacturer’s recommendations. A total of 12,570,936 raw reads, with an average length of 101 bp, were obtained during paired-end sequencing (2 × 100-bp reads) using an Illumina NovaSeq 6000 system. There were 12,170,124 reads remaining after filtering in CLC Genomics Workbench v.10 using the trim tool (quality limit = 0.03, maximum ambiguous nucleotides = 2, and minimum length = 80), which were assembled using SPAdes v.3.15.4 (3) and Unicycler v.0.4.9 (4). The initial assembly was acquired using Unicycler followed by SPAdes with the following parameters: –isolate mode with –trusted-contigs option (trusted contigs were taken from the Unicycler assembly). Contigs with a length of ≤500 bp or with coverage of ≤50× were eliminated from the final assembly. Assembly statistics were measured with QUAST v.5.0.2 (5). Estimation of completeness and contamination was performed using CheckM v.1.2.1 (6) with a bacteria-specific marker set. Genome annotation was performed using the NCBI Prokaryotic Genome Annotation Pipeline (PGAP) v.6.3 (7).

The genome assembly of strain 12-16-PSH contained 63 contigs, with a total length of 2,468,647 bp, an *N*_50_ value of 133,167 bp, and a G+C content of 35.09%. The estimated completeness and contamination of the assembly were 100% and 0%, respectively. In total, 2,526 genes were predicted, including 60 RNA genes (4 rRNA genes, 52 tRNA genes, and 4 noncoding RNA genes), 2,421 protein-coding genes, and 45 pseudogenes.

A BLASTn v.2.13.0 search (nonredundant nucleotide database, limited to type species) with the 12-16-PSH 16S rRNA gene sequence revealed that the strain is a representative of the *Lactococcus* genus; the closest species were L. lactis NBRC 100933^T^ and L. cremoris DSM 20069^T^, with sequence identity values of 100% and 99.3%, respectively. The average nucleotide identity between the L. lactis NBRC 100933 and strain 12-16-PSH genomes, calculated with pyani v.0.2.8 (8), was 98.4%, implying that strain 12-16-PSH is a member of this species. The 12-16-PSH genome search revealed 42 genes encoding glycosyl transferases, 15 of which are homologous to enzymes involved in exopolysaccharide (Eps) biosynthesis, although none of them formed *eps*-like gene clusters (9). The 12-16-PSH genome lacked the genes encoding the common *Lactococcus* bacteriocin nisin, as well as the proteins involved in its processing. In turn, the genes for six homologs of lactococcins (A, B, and G type) were found, implying possible probiotic capacities of the strain.

### Data availability.

The whole-genome sequence was deposited in DDBJ/ENA/GenBank under the accession number JAPMIC000000000. The BioProject, BioSample, and SRA accession numbers are PRJNA903536, SAMN31807394, and SRR22378037, respectively.
